# Resilience as an indirect pathway linking perceived skill development and institutional support with job satisfaction and burnout among nurses in Saudi Arabia: A cross-sectional study

**DOI:** 10.1371/journal.pone.0345356

**Published:** 2026-10-08

**Authors:** Rayhanah R. Almutairi, Wjdan A. Almutairi, Faihan F. Alshaibany, Waleed M. Alshehri, Asrar S. Almutairi, Thurayya Eid, Ashwaq A. Almutairi, Norah M. Alyahya, Bandar S. Alharbi, Abdulaziz M. Alodhailah

**Affiliations:** 1 Community and Psychiatric Mental Health Nursing Department, College of Nursing, Princess Nourah bint Abdulrahman University, Riyadh, Saudi Arabia; 2 College of Nursing, King Saud Bin Abdulaziz University for Health Sciences (KSAU-HS), Riyadh, Saudi Arabia; 3 King Abdullah International Medical Research Center, Riyadh, Saudi Arabia; 4 Ministry of National Guard Health Affairs, Riyadh, Saudi Arabia; 5 Department of Nursing Administration and Education, College of Nursing, King Saud University, Riyadh, Saudi Arabia; 6 Department of Medical-Surgical Nursing, College of Nursing, King Saud University, Riyadh, Saudi Arabia; 7 Monash Nursing and Midwifery, Monash University, Melbourne, Victoria, Australia; 8 Department of Community and Psychiatric Mental Health Nursing, College of Nursing, King Saud University, Riyadh, Saudi Arabia; College of Nursing, Qassim University, SAUDI ARABIA

## Abstract

Burnout among nurses threatens healthcare quality and workforce sustainability. This study examined associations between perceived skill development, institutional support, resilience, job satisfaction, and burnout among nurses in Saudi Arabia, testing resilience as a mediator linking organizational resources to outcomes. In this cross-sectional study, 190 registered nurses from three hospitals in Riyadh completed validated instruments: Professional Development Scale in Nursing, Survey of Perceived Organizational Support, Connor–Davidson Resilience Scale-25, Index of Work Satisfaction, and Copenhagen Burnout Inventory. Hierarchical regression and regression-based mediation (PROCESS Model 4; 5,000 bootstrap samples) were conducted, controlling for age, gender, experience, hospital type, and nationality. Reporting follows STROBE guidelines. Skill development (β = 0.38, p < 0.001) and institutional support (β = 0.34, p < 0.001) were independently associated with resilience (R^2^ = 0.412). Resilience was positively associated with job satisfaction (r = 0.48) and negatively with burnout (r = −0.54). Indirect effects via resilience were statistically significant for the paths from skill development to job satisfaction (0.15,95% CI [0.09,0.22]), skill development to burnout (−0.23,95% CI [−0.32, −0.15]), institutional support to job satisfaction (0.14,95% CI [0.09,0.21]), and institutional support to burnout (−0.19,95% CI [−0.28, −0.12]). Nationality was not significantly associated with resilience (β = 0.03, p = 0.660). Higher perceived skill development and institutional support were associated with higher resilience, which in turn correlated with improved job satisfaction and reduced burnout. These findings suggest that strengthening organizational resources is associated with higher nurse resilience and, in turn, with more favorable job satisfaction and burnout profiles; given the cross-sectional design, causal or directional claims cannot be made.

## Introduction

Burnout among nurses is a persistent global challenge linked to reduced workforce retention and compromised patient care quality. Defined as a state of prolonged physical and psychological exhaustion arising from chronic occupational stress, burnout is estimated to affect 35–54% of nurses worldwide [2]. Burnout has been linked to a range of adverse outcomes, including increased patient mortality, medication errors, hospital-acquired infections, and instances of “missed care,” in which essential nursing activities are delayed or omitted under sustained time constraints and resource limitations [1,2]. Large-scale evidence indicates that each additional patient per nurse is associated with a 7% increase in the odds of patient mortality [1]. The COVID-19 pandemic further intensified these challenges, accelerating workforce attrition and exposing systemic vulnerabilities in healthcare organizations across all income settings [3].

Resilience, conceptualized as positive adaptation despite significant adversity, has consistently been associated with lower burnout and higher job satisfaction among nurses [4,5]. However, resilience is frequently framed as an individual characteristic, which risks shifting responsibility to nurses for managing systemic stressors while underemphasizing the organizational determinants that may enable resilience to develop and be sustained [3,6]. This study therefore focuses on two organizational resources that are potentially actionable through policy and leadership: perceived opportunities for professional skill development and perceived institutional support.

## Theoretical framework

This study integrates two complementary theoretical perspectives: Conservation of Resources (COR) theory [7,8] and the Job Demands–Resources (JD-R) model [9,10]. Although these frameworks share conceptual ground, they offer distinct lenses that together provide a richer basis for understanding the hypothesized relationships.

Conservation of Resources (COR) theory. COR theory posits that psychological stress occurs when valued resources are threatened, lost, or insufficient to meet environmental demands [8]. Resources encompass tangible assets (e.g., equipment), conditional resources (e.g., employment security), personal characteristics (e.g., self-efficacy, resilience), and energies (e.g., time, knowledge). A fundamental tenet is the “primacy of loss,” which holds that resource loss is disproportionately more psychologically impactful than equivalent resource gain [7]. Within this framework, resilience functions both as a personal resource, the capacity to recover from adversity, and as an outcome of successful resource accumulation. When nurses perceive adequate organizational investments in skill development and institutional support, they may engage in “resource gain spirals,” whereby initial resource gains facilitate the acquisition of additional resources, thereby enhancing resilience capacity [7]. Conversely, perceived resource deficits may trigger “loss spirals,” wherein diminished resilience accelerates burnout and further resource depletion. This dynamic is particularly relevant to understanding the inverse associations with burnout observed in this study.

Job Demands–Resources (JD-R) model. The JD-R model proposes a dual-pathway framework linking work characteristics to employee outcomes [9,10]. Job demands are physical, psychological, social, or organizational aspects of work requiring sustained effort and associated with physiological or psychological costs (e.g., workload, emotional demands, time pressure). Job resources are aspects that facilitate goal attainment, reduce demand-related costs, and stimulate personal growth (e.g., autonomy, feedback, professional development, social support). The model posits two pathways: (1) a health-impairment pathway, where excessive demands deplete energy and lead to burnout; and (2) a motivational pathway, where adequate resources foster engagement and job satisfaction. Critically, resources are theorized to be most effective when demands are high, serving as buffers against adverse effects of demanding work environments [10,11].

Theoretical integration. In this study, COR theory is applied to explain the psychological processes underlying resilience development; specifically, how organizational investments in skill development and institutional support may contribute to resource gain spirals that build the personal resource of resilience. The JD-R model provides a framework for interpreting the observed resource-side associations with job satisfaction and burnout. Because job demands were not measured, this study did not test the demand-driven health-impairment pathway, demand–resource interactions, or buffering propositions. COR theory explains why organizational resources may be associated with resilience, whereas JD-R theory provides a resource-oriented framework for interpreting the observed outcome associations.

### Research context: Saudi Arabian healthcare

Saudi Arabia’s healthcare system is undergoing substantial transformation under Vision 2030 reforms, with nursing workforce development and Saudization of the healthcare sector prioritized [12]. The nursing workforce is highly multicultural, comprising approximately 60% expatriate nurses from diverse backgrounds including the Philippines, India, and Egypt [13]. This creates complex organizational dynamics, including communication challenges, cultural adaptation stressors, and differential social support access, that distinguish this setting from more culturally homogeneous contexts. Recent studies indicate burnout prevalence rates of 48–71% among nurses in Saudi Arabia, associated with high workload, inadequate staffing, and limited professional development opportunities [14]. However, resilience research within Saudi nursing remains limited, constraining evidence-based policy development tailored to this unique healthcare environment.

### Research gap and study objectives

Despite growing recognition of resilience’s importance in nursing, critical gaps persist in understanding how organizational factors contribute to resilience and how resilience in turn relates to nurse outcomes. Few studies have examined these relationships within the Saudi Arabian healthcare context, and even fewer have employed burnout measures that distinguish between personal, work-related, and client-related dimensions of exhaustion. This study addresses these gaps by employing the Copenhagen Burnout Inventory (CBI), which conceptualizes burnout specifically as prolonged physical and psychological exhaustion rather than the Maslach triad of emotional exhaustion, depersonalization, and reduced personal accomplishment [15]. The CBI’s domain-specific approach offers nuanced insights into where organizational interventions may be most relevant.

#### Study objectives.

This study aimed to: (1) examine associations between perceived skill development and institutional support with resilience; (2) examine associations of resilience with job satisfaction and burnout; and (3) test whether resilience shows indirect effects linking organizational resources with job satisfaction and burnout (CBI total and subscales).

#### Study hypotheses.

Based on the theoretical framework and existing empirical evidence, the following directional hypotheses were formulated:

- H1: Perceived skill development is positively associated with resilience.
- H2: Perceived institutional support is positively associated with resilience.
- H3a: Resilience is positively associated with job satisfaction.
- H3b: Resilience is negatively associated with burnout (CBI total and subscale scores).
- H4: Resilience shows an indirect effect linking skill development with job satisfaction.
- H5: Resilience shows an indirect effect linking skill development with burnout.
- H6: Resilience shows an indirect effect linking institutional support with job satisfaction.
- H7: Resilience shows an indirect effect linking institutional support with burnout.

## Literature review

Resilience in contemporary nursing practice. Recent theoretical developments emphasize resilience as a multidimensional construct encompassing cognitive flexibility, emotional regulation, spiritual grounding, and social connectedness [6]. Contemporary research increasingly frames resilience as a dynamic, context-dependent process rather than a fixed individual trait, underscoring the significant influence of organizational factors on resilience development and maintenance [3]. Resilient nurses have been shown to report lower anxiety, depression, and post-traumatic stress symptoms during pandemic conditions compared to less resilient counterparts [16]. From a COR perspective, these findings suggest that resilience functions as a key personal resource enabling nurses to navigate resource-threatening environments without entering loss spirals.

Professional development as a resource for resilience. Professional development encompasses multifaceted learning experiences that enhance clinical competencies, critical thinking, emotional intelligence, and reflective practice [17]. Systematic reviews identify structured mentorship, simulation-based learning, interdisciplinary collaboration, and reflective practice sessions as key components [18]. Evidence demonstrates that nurses with greater professional development access report higher self-efficacy, lower emotional exhaustion, and enhanced job satisfaction [19]. Through a COR lens, professional development contributes cognitive resources (enhanced appraisal and coping repertoires), social resources (mentorship and peer networks), and mastery experiences (confidence through successful skill application), which together may initiate upward gain spirals that enhance resilience.

Institutional support systems. Perceived organizational support (POS) reflects employees’ global beliefs concerning organizational care and commitment to their well-being [20]. Broader reviews of the nurse work environment similarly link supportive workplace conditions to improved nurse outcomes [21]. Meta-analytic findings demonstrate robust relationships between POS and reduced burnout (ρ = −0.54), enhanced job satisfaction (ρ = 0.67), and decreased turnover intentions (ρ = −0.58) [22]. Multi-national pandemic studies revealed that nurses reporting higher organizational support exhibited greater resilience (OR = 2.84, 95% CI [2.31, 3.49]) and lower burnout (OR = 0.43, 95% CI [0.35, 0.53]) [23]. Within the JD-R model, these findings position institutional support as a key job resource operating through both motivational and health-protective pathways, a relationship that may be particularly consequential when demands are high.

Cultural considerations in a multicultural workforce. Healthcare systems in the Middle East operate within cultural contexts that influence professional relationships and organizational dynamics. Because the Saudi nursing workforce is highly multicultural, organizational support interventions must be accessible and culturally inclusive, addressing system-wide factors rather than assuming cultural homogeneity. Expatriate nurses may experience unique stressors related to cultural adaptation, language barriers, and separation from family support networks, further underscoring the need for organizational-level approaches [13,24].

## Methods

### Study design and reporting

This was a cross-sectional correlational study. Reporting follows the Strengthening the Reporting of Observational Studies in Epidemiology (STROBE) guidelines for observational studies [25] (S1 Checklist).

### Setting

Participants were nurses working in Riyadh, Saudi Arabia, recruited from three hospitals: one public and two private hospitals.

### Participants

Eligibility criteria. Registered nurses in direct patient-care roles, employed for at least six months in their current position, with English proficiency sufficient to complete the survey. English proficiency was self-reported at eligibility screening; no formal language-comprehension test or objective proficiency assessment was administered (see Limitations).

Exclusion criteria. Nurses holding administrative or education-only roles without patient-care responsibilities; surveys with more than 15% missing data.

Sampling and recruitment. A non-probability convenience sampling approach was used because a complete sampling frame was not available across institutions. Recruitment comprised two dissemination streams:

1. Closed invitation route (enumerated). Invitations were distributed via WhatsApp rosters across three Riyadh hospitals (one public, two private), yielding an enumerated denominator of 245 invitations.
2. Open-link dissemination (non-enumerated). The survey link was shared in nursing social-media groups and via snowball forwarding through professional networks. Because the number of individuals who received the link was unknown, a response rate could not be calculated for this stream.

While the initial protocol aimed for stratified sampling to ensure representation across hospital sector, clinical department, and experience level, the final approach was non-probabilistic. This methodological choice is acknowledged as a limitation affecting generalizability.

### Sample size

An a priori calculation using G*Power 3.1.9.7 for multiple regression (medium effect f^2^ = 0.15; α = 0.05; power = 0.80; five predictors) indicated a minimum of 164 participants. The target was increased to 220 to accommodate potential attrition. The final analytic sample comprised 190 complete cases, which is adequate for the primary regression and PROCESS mediation analyses but may be insufficient for robust psychometric analyses of the multi-item instruments (e.g., the 44-item IWS yields an approximately 4.3:1 participant-to-item ratio, below the 5:1 minimum recommended for factor-analytic stability [26]). This limitation is discussed further below.

### Measures and instrumentation

All instruments were administered in English. Given that 60% of the sample comprised expatriate nurses and that English is widely used as a professional language in Saudi healthcare settings, English-language administration was deemed appropriate. However, this approach may have introduced selection bias by potentially excluding nurses with limited English proficiency (see Limitations). The complete survey instruments, along with their detailed scoring procedures and interpretation guidelines, are available in S3 File.

Professional Development Scale in Nursing (PDS-N). Skill development was assessed using the PDS-N (20 items), measuring perceived access to training opportunities, mentorship programs, career advancement pathways, and continuing education resources. Items employ 5-point Likert scales (1 = *strongly disagree* to 5 = *strongly agree*). The scale demonstrated excellent internal consistency in the present sample (α = 0.91). The PDS-N was originally developed by Nolan et al. [18] and has been used in prior nursing workforce studies examining professional development and continuing education.

Survey of Perceived Organizational Support (SPOS). Institutional support was measured using a 10-item short form of the SPOS [20,27]. Item wording was adapted only to identify the hospital as the employing organization; no separately validated healthcare-specific version of the scale was used. Items assess organizational valuing of contributions, care about well-being, goal consideration, help availability, and pride in accomplishments. The response format used a 5-point Likert scale (1 = *strongly disagree* to 5 = *strongly agree*). Internal consistency was excellent (α = 0.94). The SPOS was originally developed by Eisenberger et al. [28], and the 10-item version used here follows the recommendations of Shanock et al. [27], who identified high-loading items capturing both halves of the POS definition (“valuing contributions” and “caring about well-being”). Items are freely available for research use (see S3 File for full item content).

Connor–Davidson Resilience Scale (CD-RISC-25). Resilience was evaluated using the CD-RISC-25, a 25-item self-report measure assessing personal competence, trust in one’s instincts, positive acceptance of change, control, and spiritual influences [4]. Items employ a 5-point scale (0 = *not true at all* to 4 = *true nearly all of the time*). For comparability across measures, responses were linearly transformed to a 1–5 metric using x’ = x + 1; scale scores were computed as the mean of transformed items. The CD-RISC-25 has demonstrated sound psychometric properties including good internal consistency (α = 0.89 in general population samples [4]) and test–retest reliability (ICC = 0.87) [4]. An Arabic-language version of the CD-RISC-25 has been validated in an Arabic-speaking population [29], although the English-language version was administered in the present study. In the present sample, α = 0.89.

Index of Work Satisfaction (IWS). Job satisfaction was assessed using the IWS (Part B: Attitude Questionnaire), a 44-item instrument examining satisfaction across six components: pay, autonomy, task requirements, organizational policies, professional interaction, and professional status [19]. Items are rated on a 7-point scale (1 = *strongly agree* to 7 = *strongly disagree*). Negatively worded items (e.g., items 2, 4, 7, 8, 10, 12, 15, 17, 18, 20, 21, 23, 27, 28, 30, 31, 33, 35, 36, 39, 41, 44) were reverse-coded so that higher scores indicate greater satisfaction. Scores were rescaled to a 1–5 metric for consistency with other study measures. The IWS is used by permission of Paula L. Stamps, Ph.D. Internal consistency in the present sample was good (α = 0.88).

Copenhagen Burnout Inventory (CBI). Burnout was measured using the CBI, a validated 19-item instrument assessing three conceptually distinct domains: Personal Burnout (6 items; general physical and psychological exhaustion), Work-Related Burnout (7 items; exhaustion perceived as related to work), and Client-Related Burnout (6 items; exhaustion perceived as related to work with patients/clients) [15]. Items are rated on a 5-point frequency or intensity scale. Each response is converted to a score of 0, 25, 50, 75, or 100 per the CBI manual; subscale scores are computed as the mean of their constituent items, and the total score is computed as the unweighted mean of all 19 items rather than as the average of the three subscale means, yielding a 0–100 metric with higher scores indicating greater burnout. The CBI conceptualizes burnout specifically as prolonged exhaustion, distinguishing it from the Maslach triad of emotional exhaustion, depersonalization, and reduced accomplishment [15]. Internal consistency in the present sample was α = 0.89 for the total score.

### Data collection procedures

Data were collected from 31 August 2025–31 December 2025 using Google Forms. To reduce duplicate submissions, the setting “Limit to 1 response” was enabled (requiring Google sign-in). Google sign-in email addresses were excluded from the dataset exported for analysis; no identifying information was retained in the analytic file. The landing page contained a study information sheet; participants provided electronic informed consent before accessing the survey items. Estimated completion time was 10–15 minutes. To mitigate common-method bias, the survey employed procedural remedies including ensuring participant anonymity, varying response anchors across instruments (5-point, 7-point, and 0–4 scales), and separating predictor and outcome measures within the survey sequence.

### Missing data

Missingness was minimal (< 3% for any single variable) and showed no systematic patterns. Responses with > 15% missing data were excluded. Remaining analyses used complete cases (listwise deletion).

### Statistical analysis

Analyses were conducted using IBM SPSS version 29.0 with the PROCESS macro version 4.2 [30]. Analysis proceeded in three phases:

1. Preliminary analyses. Data screening for outliers and normality (skewness/kurtosis within ± 2.0); internal consistency assessment (Cronbach’s Alpha); descriptive statistics; Pearson bivariate correlations; variance inflation factors (VIF) for multicollinearity.
2. Primary analyses.
  - *Hierarchical multiple regression* testing H1–H2 (organizational resources predicting resilience). Block 1 entered demographic covariates: age, gender, clinical experience, hospital type (public/private), and nationality (Saudi/expatriate). Block 2 added skill development and institutional support. Cohen’s f^2^ was calculated for individual predictors in the final model.
  - *Pearson correlations* for H3a–H3b (resilience associations with job satisfaction and burnout).
  - *Mediation analyses* using PROCESS Model 4 with 5,000 bias-corrected bootstrap samples for H4–H7. All PROCESS models included age, gender, clinical experience, hospital type, and nationality as covariates in both mediator and outcome equations. Indirect effects were considered statistically significant if the 95% bootstrap confidence interval excluded zero.
3. Supplementary analyses. (a) Subscale mediation analyses for each CBI domain; (b) exploratory moderation testing (clinical experience × skill development → resilience).

Note on statistical approach. The PROCESS macro employs regression-based path analysis using observed variables (scale means) and does not produce global model fit indices (CFI, RMSEA, SRMR) characteristic of structural equation modeling with latent variables. Accordingly, such indices are not reported. This study did not conduct SEM.

Common-method and discriminant-validity analyses. To assess potential common-method bias, Harman’s single-factor test was conducted using an unrotated factor analysis of the item responses from the five substantive scales. The first unrotated factor accounted for 32.4% of the total variance, below the commonly used 50% screening threshold. This result suggests that a single factor did not account for most of the observed variance; however, Harman’s test is an imperfect diagnostic and does not definitively exclude common-method bias. Discriminant validity was assessed using the heterotrait–monotrait ratio (HTMT). All HTMT ratios were below 0.85, with the largest ratio equal to 0.78, supporting adequate discriminant validity among the study constructs.

### Ethical considerations

This study was approved by the King Saud University Institutional Review Board (KSU-HE #25–942; approved 19 August 2025). Informed consent was obtained electronically from all participants prior to data collection. Participation was voluntary. All procedures were conducted in accordance with the ethical standards of the institutional review board and the 1964 Helsinki Declaration and its later amendments.

## Results

### Participant flow and sample characteristics

For the closed invitation route (WhatsApp rosters across three Riyadh hospitals), 245 invitations were distributed and 210 nurses initiated the survey (contact-to-initiation rate: 85.7%). Overall, 210 individuals initiated the survey and 190 provided complete responses suitable for analysis (completion rate among initiators: 90.5%). A response rate could not be calculated for the open-link dissemination because the number of individuals who received the link was unknown. The participant flow is depicted in S2 Fig.

Sample characteristics are summarized in Table 1. The sample included 114 female nurses (60.0%) and 76 male nurses (40.0%), with a mean age of 35.2 years (SD = 8.9) and mean clinical experience of 10.4 years (SD = 7.1). Most held a bachelor’s degree (70.5%). Emergency departments represented the largest clinical area (40.0%), and public hospital employment was predominant (64.7%). Expatriate nurses constituted 60.0% of the sample (n = 114). Nationality figures are percentages of the total sample rather than of the expatriate subgroup: the Philippines 32.1% (n = 61), India 15.8% (n = 30), and Egypt 12.1% (n = 23). These three nationalities together accounted for all expatriate participants; no other national groups were represented (see Limitations).

**Table 1:** Participant characteristics (N = 190).

Characteristic	n (%)
**Gender**	
Female	114 (60.0)
Male	76 (40.0)
**Age group**	
21–30 years	47 (24.7)
31–40 years	85 (44.7)
41–50 years	38 (20.0)
> 50 years	20 (10.5)
**Clinical experience**	
≤ 5 years	38 (20.0)
6–10 years	66 (34.7)
11–15 years	47 (24.7)
> 15 years	38 (20.0)
**Education level**	
Diploma	32 (16.8)
Bachelor’s	134 (70.5)
Master’s	24 (12.6)
**Department**	
General ward	57 (30.0)
Emergency	76 (40.0)
ICU	57 (30.0)
**Hospital type**	
Public	123 (64.7)
Private	67 (35.3)
**Nationality**	
Saudi	76 (40.0)
Expatriate	114 (60.0)

### Descriptive statistics, reliability, and correlations

Scale descriptive statistics, internal consistency coefficients, and bivariate Pearson correlations are presented in Table 2. All measures demonstrated acceptable to excellent internal consistency (α = 0.88–0.94). Variable distributions were approximately normal (skewness and kurtosis within ± 2.0 for all variables).

**Table 2:** Descriptive statistics and bivariate correlations (N = 190).

Variable	M (SD)	α	1	2	3	4	5
1. Skill development	3.45 (0.82)	0.91	—				
2. Institutional support	3.31 (0.88)	0.94	.49***	—			
3. Resilience	3.58 (0.75)	0.89	.55***	.51***	—		
4. Job satisfaction	3.37 (0.84)	0.88	.42***	.45***	.48***	—	
5. Burnout (CBI total)	52.4 (18.7)	0.89	−.41***	−.45***	−.54***	−.59***	—

*Note.* *** *p* < .001. CBI scores are reported on the 0–100 scale.

Mean CBI subscale scores were 54.2 (SD = 19.3) for personal burnout, 51.8 (SD = 18.2) for work-related burnout, and 51.1 (SD = 19.5) for client-related burnout. Because the CBI does not have universally validated diagnostic cutoffs applicable across populations [15], scores are interpreted as continuous outcomes. Descriptive distributional information is provided in S3 File for contextual reference.

Correlation analyses supported the hypothesized relationships: skill development and institutional support were positively and significantly associated with resilience (*r* = .55 and *r* = .51, respectively; *p* < .001), supporting H1 and H2. Resilience was positively associated with job satisfaction (*r* = .48, *p* < .001; supporting H3a) and negatively associated with burnout (*r* = −.54, *p* < .001; supporting H3b).

### Organizational resources predicting resilience

Hierarchical multiple regression results are summarized in Table 3. Model 1, including demographic covariates, explained a small and non-significant proportion of variance in resilience (R^2^ = .030, *p* = .341). Adding organizational resources substantially increased explained variance (ΔR^2^ = .382, *p* < .001), with the full model accounting for 41.2% of resilience variance. Both skill development (β = .38, *p* < .001, f^2^ = 0.15) and institutional support (β = .34, *p* < .001, f^2^ = 0.11) emerged as significant independent predictors, with Cohen’s f^2^ values of 0.15 and 0.11, respectively. The corresponding partial eta-squared values, calculated as η^2^ = f^2^/(1 + f^2^), were .130 for skill development and .099 for institutional support [31]. Nationality was not significantly associated with resilience when organizational resources were controlled (β = .03, *p* = .660), suggesting that the associations between organizational resources and resilience were consistent across Saudi and expatriate nurses. Variance inflation factors were below 2.1 for all predictors, indicating no multicollinearity concerns.

**Table 3:** Hierarchical multiple regression predicting resilience (N = 190).

Predictor	B	SE	β	t	p	95% CI	f^2^
**Model 1: Demographics**							
Constant	3.18	0.30	—	10.60	<.001	[2.59, 3.77]	
Age	0.07	0.06	.08	1.17	.244	[−0.05, 0.19]	0.01
Gender	0.11	0.12	.07	0.92	.359	[−0.13, 0.35]	0.00
Experience	0.09	0.07	.09	1.29	.199	[−0.05, 0.23]	0.01
Hospital type	−0.08	0.13	−.05	−0.62	.536	[−0.34, 0.18]	0.00
Nationality	0.05	0.11	.03	0.45	.652	[−0.17, 0.27]	0.00
**Model 2: + Organizational resources**							
Constant	1.35	0.23	—	5.87	<.001	[0.90, 1.80]	
Age	0.01	0.05	.01	0.20	.842	[−0.09, 0.11]	0.00
Gender	0.06	0.10	.04	0.60	.549	[−0.14, 0.26]	0.00
Experience	0.03	0.06	.03	0.50	.618	[−0.09, 0.15]	0.00
Hospital type	−0.11	0.10	−.07	−1.10	.273	[−0.31, 0.09]	0.01
Nationality	0.04	0.09	.03	0.44	.660	[−0.14, 0.22]	0.00
**Skill development**	**0.35**	**0.06**	**.38**	**5.83**	**<.001**	**[0.23, 0.47]**	**0.15**
**Institutional support**	**0.29**	**0.06**	**.34**	**4.83**	**<.001**	**[0.17, 0.41]**	**0.11**

*Note.* Model 1: R^2^ = .030, F(5, 184) = 1.14, *p* = .341; Model 2: R^2^ = .412, F(7, 182) = 18.32, *p* < .001; ΔR^2^ = .382, *p* < .001.

### Mediation analyses

Regression-based mediation results (PROCESS Model 4) are presented in Table 4. All PROCESS models included age, gender, clinical experience, hospital type, and nationality as covariates in both mediator (resilience) and outcome (job satisfaction or burnout) equations.

**Table 4:** Mediation analysis results (PROCESS Model 4; N = 190).

Pathway	Direct effect (c’)	Indirect effect (ab)	95% Bootstrap CI	Total effect (c)	PM (%)
**SD → Resilience → JS**					
a path (SD → Resilience)	0.38***	—	[0.25, 0.51]	—	—
b path (Resilience → JS)	0.39***	—	[0.26, 0.52]	—	—
Direct (c’)	0.27**	—	[0.12, 0.42]	—	—
Indirect	—	0.15	[0.09, 0.22]	0.42***	35.7
**SD → Resilience → BO**					
a path (SD → Resilience)	0.38***	—	[0.25, 0.51]	—	—
b path (Resilience → BO)	−0.61***	—	[−0.76, −0.46]	—	—
Direct (c’)	−0.17*	—	[−0.33, −0.01]	—	—
Indirect	—	−0.23	[−0.32, −0.15]	−0.40***	58.2
**IS → Resilience → JS**					
a path (IS → Resilience)	0.34***	—	[0.21, 0.47]	—	—
b path (Resilience → JS)	0.41***	—	[0.28, 0.54]	—	—
Direct (c’)	0.31***	—	[0.17, 0.45]	—	—
Indirect	—	0.14	[0.09, 0.21]	0.45***	31.1
**IS → Resilience → BO**					
a path (IS → Resilience)	0.34***	—	[0.21, 0.47]	—	—
b path (Resilience → BO)	−0.56***	—	[−0.71, −0.41]	—	—
Direct (c’)	−0.26**	—	[−0.42, −0.10]	—	—
Indirect	—	−0.19	[−0.28, −0.12]	−0.45***	42.2

*Note.* * *p* < .05, ** *p* < .01, *** *p* < .001. SD = Skill Development; IS = Institutional Support; JS = Job Satisfaction; BO = Burnout (CBI total); PM = Proportion Mediated. CIs are 95% bias-corrected bootstrap intervals (5,000 samples). All models included demographic covariates. Negative burnout coefficients indicate higher resilience associated with lower burnout.

The skill development → burnout pathway showed the strongest indirect effect, with 58.2% of the total effect operating through resilience. Harman’s single-factor test indicated that the first unrotated factor accounted for 32.4% of the total variance, below the commonly used 50% screening threshold. The HTMT analysis found that all ratios were below 0.85, with a maximum of 0.78, supporting adequate discriminant validity among the study constructs. These results reduce, but do not eliminate, concerns about common-method variance and construct overlap; the indirect effects should therefore be interpreted as statistically supported association patterns rather than definitive causal mechanisms.

### Burnout subscale analyses

Supplementary mediation analyses for each CBI subscale are presented in Table 5. Resilience showed the strongest indirect effects for personal burnout, followed by work-related and client-related burnout. These results suggest that resilience may be particularly associated with protection against general physical and psychological exhaustion, while other factors (e.g., specific patient-interaction skills, staffing levels, workload structures) may play relatively larger roles in client-related burnout.

**Table 5:** Indirect effects by CBI burnout subscale (PROCESS Model 4; N = 190).

Pathway	Personal burnout	Work-related burnout	Client-related burnout
**Skill development → Resilience → Burnout**			
Indirect effect	−0.26***	−0.22***	−0.18***
95% CI	[−0.36, −0.17]	[−0.31, −0.14]	[−0.27, −0.10]
Proportion mediated	62.4%	55.8%	49.3%
**Institutional support → Resilience → Burnout**			
Indirect effect	−0.22***	−0.18***	−0.15***
95% CI	[−0.31, −0.14]	[−0.27, −0.11]	[−0.24, −0.08]
Proportion mediated	48.7%	40.5%	36.2%

*Note.* *** *p* < .001. All models included demographic covariates.

### Supplementary analysis: Experience as a moderator

Exploratory moderation analysis examined whether clinical experience moderated the skill development–resilience association. The interaction term was not statistically significant (β = 0.08, *p* = .312), indicating that the positive association between skill development and resilience was consistent across nurses with varying clinical experience levels. Given the non-significant interaction, moderated mediation (PROCESS Model 7) was not pursued.

### Summary of hypothesis testing

The statistical analysis provided a comprehensive evaluation of the proposed theoretical model. As summarized in Table 6, the predicted directions for H1–H7 were supported by the reported regression coefficients, correlations, and bootstrap confidence intervals; mediation hypotheses are evaluated using indirect effects and confidence intervals rather than a single omnibus p-value. Specifically, the results indicated that both skill development and institutional support were significant independent predictors of nurse resilience.

**Table 6:** Summary of hypothesis testing.

Hypothesis	Description	Result
H1	Skill development positively associated with resilience	Supported (β = .38, *p* < .001)
H2	Institutional support positively associated with resilience	Supported (β = .34, *p* < .001)
H3a	Resilience positively associated with job satisfaction	Supported (*r* = .48, *p* < .001)
H3b	Resilience negatively associated with burnout	Supported (*r* = −.54, *p* < .001)
H4	Indirect effect: SD → Resilience → JS	Supported (PM = 35.7%)
H5	Indirect effect: SD → Resilience → BO	Supported (PM = 58.2%)
H6	Indirect effect: IS → Resilience → JS	Supported (PM = 31.1%)
H7	Indirect effect: IS → Resilience → BO	Supported (PM = 42.2%)

Furthermore, the mediation analyses indicated that resilience operated as a statistical intervening variable in the associations between these organizational resources and nurse outcomes. The proportion mediated (PM) was high for the burnout pathways, with 58.2% of the association between skill development and burnout accounted for by resilience. These findings indicate that much of the association between organizational resources and both burnout and job satisfaction is shared with resilience, although the cross-sectional design does not permit conclusions about directionality. Overall, the model accounted for a substantial share of the variance in resilience among nurses in this Saudi Arabian acute care sample.

## Discussion

### Summary of key findings

This study examined associations between organizational resources (skill development and institutional support), resilience, job satisfaction, and burnout among nurses working in Saudi Arabia, employing the Copenhagen Burnout Inventory to assess burnout as multidimensional exhaustion. The findings provide empirical support for all seven hypotheses: both skill development and institutional support were significantly and independently associated with higher resilience; resilience was positively associated with job satisfaction and negatively associated with burnout; and resilience showed statistically significant indirect effects linking organizational resources with nurse outcomes, with particularly strong effects for the burnout pathways.

### Theoretical implications

The findings are consistent with Conservation of Resources theory and with the resource-oriented (motivational) side of the Job Demands–Resources model. Because job demands were not measured, the findings speak only to the main-effect contribution of resources and do not constitute a full test of the JD-R model’s dual-pathway or demand-buffering propositions. Consistent with COR theory’s principle of resource investment, nurses who perceived greater organizational investments in their professional development and institutional support demonstrated higher resilience, a pattern consistent with “resource gain spirals” whereby organizational resources contribute to the accumulation of personal resources [8]. The substantial variance in resilience explained by organizational factors (ΔR2 = .382) underscores that resilience, while often conceptualized as an individual characteristic, appears to be substantially influenced by organizational context. COR theory’s “primacy of loss” is also relevant for interpreting the inverse associations with burnout: when organizational resources are perceived as insufficient, nurses may be more vulnerable to loss spirals, experiencing diminished resilience that accelerates exhaustion and further erodes their capacity to acquire or protect remaining resources. The stronger indirect effects observed for burnout compared to job satisfaction are consistent with this asymmetry, as burnout may be particularly sensitive to resource depletion dynamics.

From a JD-R perspective, the mediation findings are most directly consistent with the motivational pathway: skill development and institutional support (job resources) were associated with higher resilience, which in turn was associated with greater job satisfaction and with lower burnout. The particularly strong indirect effect observed for the burnout pathway (58.2% of the skill development–burnout association) should be interpreted as a resource-side statistical association. Because job demands were not measured, the demand-driven health-impairment pathway and the JD-R demand–resource interaction were not tested.

The differential pattern of indirect effects across CBI subscales provides additional theoretical insights. Resilience showed the strongest associations with personal burnout, which refers to generalized exhaustion experienced regardless of work context, followed by work-related and client-related burnout. This gradient suggests that while organizational resources may be associated with resilience that broadly relates to protection against exhaustion, additional mechanisms (specific clinical skills, staffing adequacy, patient interaction training) may be needed to address the demands specific to direct patient care. This interpretation aligns with COR theory’s emphasis on resource specificity: different types of demands may require qualitatively different types of resources for effective coping [8].

### Comparison with previous research

The observed association between resilience and burnout (r = −.54) is consistent in direction with prior nursing research [5], and the associations between organizational resources and resilience are consistent with prior research demonstrating that supportive organizational environments are associated with higher nurse resilience [3,7]. However, the present study extends previous research in three ways. First, the use of the CBI, which conceptualizes burnout specifically as exhaustion rather than the Maslach triad, provides a focused examination of how resilience relates to the core experience of burnout across distinct domains. Second, by simultaneously examining skill development and institutional support as independent predictors, the findings demonstrate that both types of organizational resources may make unique contributions to resilience. Third, the inclusion of a multicultural nursing workforce (60% expatriate) extends the evidence base beyond Western and culturally homogeneous samples.

Several recent Saudi studies have examined resilience and burnout in overlapping populations, and the present findings are best read alongside them. Siraj et al. [32] surveyed 1,174 healthcare professionals nationally and reported that burnout was associated with lower resilience and weaker supervisory support, but assessed burnout with the Maslach Burnout Inventory and resilience with the 10-item CD-RISC, treated resilience as a correlate rather than an intervening variable, and sampled several professional groups rather than nurses in direct patient care. Jaber et al. [33] surveyed 1,747 nurses across Riyadh hospitals and primary care centres and interviewed 90 of them, identifying workload, staffing, and leadership as burnout triggers and cataloguing self-coping strategies, but did not measure resilience with a standardised instrument or model organizational resources as antecedents. The present study differs in three respects: skill development and institutional support are measured as distinct organizational resources rather than as a single work-environment construct; resilience is positioned as an intervening variable, with indirect effects quantified using bootstrapped confidence intervals; and burnout is assessed with the CBI, which separates personal, work-related, and client-related exhaustion.

The finding that nationality was not significantly associated with resilience when organizational resources were controlled is noteworthy. It suggests that the associations between organizational support and resilience may operate similarly across diverse cultural backgrounds. However, this should be interpreted cautiously given the cross-sectional design and the possibility of unmeasured confounders (e.g., differential exposure to job demands across nationality groups).

### Practical implications

The results suggest several potentially actionable implications for healthcare organizations and nursing leaders seeking to enhance workforce sustainability. First, the significant and independent contributions of both skill development and institutional support suggest that comprehensive approaches combining professional development with broader organizational support systems may be more effective than addressing either dimension in isolation. In practice, this could involve structured professional development pathways (mentorship, simulation-based training, continuing education) paired with organizational-level changes signaling institutional care (fair policies, adequate resourcing, recognition systems, transparent leadership communication). Second, the strong indirect effects, particularly for burnout, suggest that interventions explicitly targeting resilience development may yield meaningful returns. Evidence-based resilience programs incorporating mindfulness training, cognitive restructuring, and social support enhancement have demonstrated feasibility and acceptability in nursing populations [34] and could be integrated with professional development initiatives. Third, the differential indirect effects across CBI domains suggest that while resilience-building may be particularly relevant for addressing personal and work-related burnout, client-related burnout may require additional interventions targeting the specific demands of patient care. Such interventions might include communication skills training, structured debriefing following difficult patient encounters, and structural reforms to workload and staffing. Finally, given the multicultural composition of the workforce, organizational support systems should be designed with cultural inclusivity by ensuring language accessibility in development programs, offering culturally informed peer support networks, and attending to the unique stressors experienced by expatriate workers (e.g., cultural adaptation, separation from family). The absence of a nationality effect on resilience once organizational resources were accounted for suggests that system-wide organizational measures are a reasonable starting point in multicultural workforces, provided they are delivered in ways accessible to nurses of all national backgrounds.

### Strengths and limitations

This study has several strengths. The theory-driven integration of COR and JD-R perspectives provides a coherent framework for understanding organizational correlates of resilience and nurse outcomes. The use of the CBI’s domain-specific approach reveals differentiated associations across burnout dimensions that have practical implications for targeted intervention. The analytic approach applied bootstrapped confidence intervals for indirect effects and reported Cohen’s f2 effect sizes for key predictors, supporting more informative inference than p-values alone. The inclusion of a multicultural nursing workforce (60% expatriate) extends the evidence base to a context that is increasingly common in healthcare systems globally.

Several limitations should be considered when interpreting these findings. First, the cross-sectional design precludes causal or directional inference; the observed associations among organizational resources, resilience, and nurse outcomes may be reciprocal, and longitudinal or experimental designs are needed to establish temporal precedence. Second, the non-probability convenience sampling strategy, combined with an open-link dissemination stream for which a sampling frame and response rate could not be established, limits generalizability; nurses who chose to participate through informal social-media and snowball channels may differ systematically from non-participants, and the stratified sampling planned in the original protocol was not achieved; in addition, all expatriate participants belonged to three national groups (the Philippines, India, and Egypt), so the findings may not extend to expatriate nurses from other backgrounds. Third, all measures relied on self-report, which introduces potential common-method variance and social-desirability bias; procedural remedies (anonymity, varied response anchors, measurement separation) and a post-hoc Harman’s single-factor test were applied and did not indicate that a single factor dominated the covariance structure, but these are imperfect safeguards and residual common-method bias cannot be excluded. Fourth, the English-only administration of all instruments, in a workforce where 60% of participants were expatriate nurses from diverse first-language backgrounds, may have introduced measurement inequivalence and selection bias by excluding nurses with limited English proficiency; formal measurement-invariance testing across language or nationality subgroups was not conducted and is an important direction for future validation work. Fifth, although the final analytic sample (N = 190) was adequate for the primary regression and mediation analyses, it was underpowered for confirmatory psychometric evaluation of the longer instruments (e.g., the 44-item IWS), and reliance on Cronbach’s alpha alone, without confirmatory factor analysis, limits the strength of conclusions about construct validity. Finally, despite the HTMT results supporting adequate discriminant validity between resilience and burnout in this sample, the Copenhagen Burnout Inventory’s exhaustion-focused conceptualization of burnout shares conceptual ground with resilience as commonly operationalized, and the particularly strong indirect effects observed for the burnout pathways should be interpreted with this potential conceptual overlap in mind.

In the post-hoc Harman’s single-factor test, the first unrotated factor accounted for 32.4% of the total variance, below the commonly used 50% screening threshold, indicating that a single factor was unlikely to account for most of the observed variance. This result is reassuring but not definitive, and residual common-method bias cannot be ruled out.

### Recommendations for future research

Finally, although the HTMT results support discriminant validity at the prespecified screening threshold, future studies should replicate these assessments using confirmatory factor analysis and longitudinal or multi-source designs to reduce uncertainty about construct overlap and temporal ordering.

## Conclusion

This study provides evidence that perceived skill development and institutional support are independently associated with nurse resilience, and that resilience shows meaningful indirect associations linking these organizational resources with job satisfaction and, particularly, burnout among nurses in Saudi Arabia. The theoretical integration of Conservation of Resources theory and the resource-oriented side of the Job Demands–Resources model provided a coherent framework for interpreting these associations, while the Harman’s single-factor and HTMT analyses offered reassurance, though not definitive proof, against common-method bias and severe conceptual overlap between resilience and burnout. Given the cross-sectional design, these findings should be understood as statistically supported association patterns rather than causal mechanisms. Nonetheless, they suggest that organizational investment in structured professional development and institutional support systems may be a worthwhile focus for initiatives aiming to strengthen nurse resilience and, in turn, support workforce well-being and sustainability in multicultural Saudi Arabian healthcare settings. Future longitudinal and multi-source research is needed to confirm the directionality of these relationships and to further clarify the boundaries between resilience and burnout as distinct constructs.

## Supporting information

S1 Checklist STROBE checklist for cross-sectional studies. (DOCX)

S2 Fig Participant flow diagram. (DOCX)

S3 File Complete survey instruments and scoring procedures. (DOCX)
