# Supplementary material for: Resilience as an indirect pathway linking perceived skill development and institutional support with job satisfaction and burnout among nurses in Saudi Arabia: A cross-sectional study
Source: PLoS One. 2026 Oct 8;21(10):e0345356. doi: 10.1371/journal.pone.0345356 (PMC13648968; doi:10.1371/journal.pone.0345356)
Supplement: Supplementary file 1 [file pone.0345356.s001.docx]

**S1 Checklist. STROBE checklist for cross-sectional studies.**

Manuscript PONE-D-26-09718: “Resilience as an indirect pathway linking perceived skill development and institutional support with job satisfaction and burnout among nurses in Saudi Arabia: A cross-sectional study”

# S1 Checklist. STROBE checklist for cross-sectional studies.

| **No** | **STROBE item** | **Recommendation** | **Location** |
| --- | --- | --- | --- |
| 1 | Title and abstract | Indicate cross-sectional design | Title; Abstract |
| 2 | Background/rationale | Scientific background and rationale | Introduction, ¶1–2 |
| 3 | Objectives | State specific objectives/hypotheses | Introduction: Objectives; Hypotheses |
| 4 | Study design | Present key elements of study design | Methods: Study design |
| 5 | Setting | Describe setting, locations, relevant dates | Methods: Setting; Data collection |
| 6 | Participants | Give eligibility criteria, sources/methods of selection | Methods: Participants; Sampling |
| 7 | Variables | Define outcomes, exposures, confounders | Methods: Measures; Statistical analysis |
| 8 | Data sources | For each variable, give sources of data and assessment methods | Methods: Measures (with S3 File) |
| 9 | Bias | Describe any efforts to address potential sources | Methods: Data collection (CMB); Limitations |
| 10 | Study size | Explain how the study size was arrived at | Methods: Sample size |
| 11 | Quantitative variables | Explain how quantitative variables were handled | Methods: Measures; Analysis |
| 12 | Statistical methods | Describe all statistical methods | Methods: Statistical analysis |
| 13 | Participants (results) | Report numbers at each stage; consider flow diagram | Results: Participant flow; S2 Fig |
| 14 | Descriptive data | Give characteristics of participants | Results: Table 1 |
| 15 | Outcome data | Report numbers of outcomes | Results: Tables 2–5 |
| 16 | Main results | Give unadjusted/adjusted estimates with precision | Results: Tables 3–5 |
