# Supplementary material for: Resilience as an indirect pathway linking perceived skill development and institutional support with job satisfaction and burnout among nurses in Saudi Arabia: A cross-sectional study
Source: PLoS One. 2026 Oct 8;21(10):e0345356. doi: 10.1371/journal.pone.0345356 (PMC13648968; doi:10.1371/journal.pone.0345356)
Supplement: Supplementary file 2 [file pone.0345356.s002.docx]

# S2 Fig. Participant flow diagram.

### Inclusion criteria (met by the final analytic sample, n = 190)

- Registered nurse engaged in direct patient care
- At least 6 months of experience in current position
- Demonstrated proficiency in English
- Less than 15% missing survey data

### Exclusion criteria (applied during screening)

- Administrative-only role, no patient-care responsibilities (n = 8)
- Education-only role, no patient-care responsibilities (n = 4)
- Non-nursing healthcare staff (n = 3)
