# Supplementary material for: Resilience as an indirect pathway linking perceived skill development and institutional support with job satisfaction and burnout among nurses in Saudi Arabia: A cross-sectional study
Source: PLoS One. 2026 Oct 8;21(10):e0345356. doi: 10.1371/journal.pone.0345356 (PMC13648968; doi:10.1371/journal.pone.0345356)
Supplement: Supplementary file 3 [file pone.0345356.s003.docx]

# S3 File. Complete survey instruments and scoring procedures.

This file lists the full items used for each instrument, response formats, scoring procedures, and internal consistency (Cronbach’s alpha) in the present sample. Item wording is reproduced as administered.

## 1. Professional Development Scale in Nursing (PDS-N)

Total items: 20	|	Response format: 5-point Likert scale (1 = Strongly disagree, 2 = Disagree, 3 = Neutral, 4 = Agree, 5 = Strongly agree)

**Scoring: overall score is the mean of all 20 items.**

### Professional Development and Training Opportunities

- My workplace provides adequate opportunities for professional development.
- I have access to continuing education courses relevant to my role.
- My employer supports attendance at professional conferences and workshops.
- Tuition assistance is available for further education (certifications, degrees).
- My organization offers mentoring relationships with experienced nurses.

### Career Advancement

- There are clear pathways for career advancement in my organization.
- I believe I can be promoted based on merit and performance.
- My organization values and recognizes my professional contributions.
- I have opportunities to take on leadership roles and increased responsibilities.
- Professional development is linked to compensation and promotion decisions.

### Learning and Skill Development

- My workplace encourages development of new clinical skills and competencies.
- Regular in-service training programs are available to nursing staff.
- I have access to simulation-based learning opportunities.
- Interdisciplinary collaboration and learning are encouraged.
- Reflective practice and debriefing sessions are available after critical incidents.

### Organizational Support for Professional Growth

- My supervisor supports and encourages my professional development.
- My organization provides resources (time and funding) for skill-building.
- Clinical practice guidelines are regularly updated and accessible.
- I receive constructive feedback on my performance and areas for growth.
- My organization invests in developing nurse expertise and specialization.

**Internal consistency (present sample): Cronbach’s α = 0.91**

### Score interpretation

| **Mean Score** | **Interpretation** |
| --- | --- |
| 1.0–2.0 | Low professional development support |
| 2.1–3.0 | Moderate support |
| 3.1–4.0 | High support |
| 4.1–5.0 | Very high support |

## 2. Survey of Perceived Organizational Support (SPOS)

Total items: 16 (as originally drafted — see note above)	|	Response format: 5-point Likert scale (1 = Strongly disagree to 5 = Strongly agree)

**Scoring: overall score is the mean of all retained items.**

### Organizational Care and Support

- My hospital genuinely cares about my well-being and job satisfaction.
- My organization values my contributions to patient care.
- The hospital would be concerned about my welfare if I experienced problems.
- My organization is willing to extend itself to help me when needed.

### Fair Policies and Treatment

- My hospital treats nurses fairly and equitably.
- Organizational policies are applied consistently across staff.
- Decisions in my workplace are transparent and justified.
- I am treated with respect regardless of position or tenure.

### Recognition and Support

- My good work performance is recognized and appreciated.
- I receive acknowledgment when I perform well or exceed expectations.
- My hospital publicly recognizes nursing achievements.
- I feel valued for my clinical expertise and knowledge.

### Resource Provision, Staffing, and Professional Support

- My organization provides adequate staffing levels.
- Necessary equipment and supplies are available for patient care.
- My workplace provides a safe and supportive environment.
- My supervisors are approachable and supportive.

**Internal consistency (present sample): Cronbach’s α = 0.94**

### Score interpretation

| **Mean Score** | **Interpretation** |
| --- | --- |
| 1.0–2.0 | Low organizational support |
| 2.1–3.0 | Moderate support |
| 3.1–4.0 | High support |
| 4.1–5.0 | Very high support |

## 3. Connor–Davidson Resilience Scale (CD-RISC-25)

Total items: 25	|	Original response scale: 0–4 (0 = Not true at all, 4 = True nearly all of the time)

For comparability with the other instruments in this survey, item responses were linearly transformed to a 1–5 metric (x′ = x + 1) before scale scores were computed as the mean of transformed items.

**Internal consistency (present sample): Cronbach’s α = 0.89**

Validation: An Arabic-language version of the CD-RISC-25 has been validated (Alfuqaha & Alhalaiqa, 2023, PLOS ONE), though not specifically in a nursing population. The present study administered the scale in English; see manuscript Limitations for discussion of English-only administration.

*The 25 individual item statements are proprietary to the scale developers (Connor & Davidson, 2003) and are not reproduced here; they are available under license from the copyright holder.*

### Score interpretation

| **Mean Score** | **Interpretation** |
| --- | --- |
| 1.0–2.0 | Low resilience |
| 2.1–3.0 | Moderate resilience |
| 3.1–4.0 | High resilience |
| 4.1–5.0 | Very high resilience |

## 4. Index of Work Satisfaction (IWS, Part B – Attitude Questionnaire)

Total items: 44	|	Response format: 7-point Likert scale (1 = Strongly agree to 7 = Strongly disagree); negatively worded items were reverse-coded so that higher scores indicate greater satisfaction, then rescaled to a 1–5 metric for consistency with the other study measures.

Subscales:

- Pay (8 items)
- Autonomy (8 items)
- Task Requirements (9 items)
- Organizational Policies (6 items)
- Professional Interactions (7 items)
- Professional Status (6 items)

**Scoring: overall job satisfaction is the mean of all 44 items (after reverse-coding and rescaling).**

**Internal consistency (present sample): Cronbach’s α = 0.88**

*The IWS is used by permission of Paula L. Stamps, Ph.D. (Stamps, 1997); the 44 individual item statements are not reproduced here due to instrument licensing terms. Researchers wishing to use the IWS should contact the copyright holder.*

### Score interpretation

| **Mean Score** | **Interpretation** |
| --- | --- |
| 1.0–2.0 | Low satisfaction |
| 2.1–3.0 | Moderate satisfaction |
| 3.1–4.0 | High satisfaction |
| 4.1–5.0 | Very high satisfaction |

## 5. Copenhagen Burnout Inventory (CBI)

Total items: 19

Subscales:

- Personal Burnout (6 items)
- Work-Related Burnout (7 items)
- Client-Related Burnout (6 items)

**Scoring: each response is scored 0, 25, 50, 75, or 100 per the CBI manual; subscale and total scores are the mean of constituent items (0–100 scale, higher = greater burnout).**

**Internal consistency (present sample): Cronbach’s α = 0.89 (total score)**

*The CBI item statements are freely available for research use from the original publication (Kristensen, Borritz, Villadsen & Christensen, 2005) and are not reproduced here for brevity.*

### Score interpretation

| **Score** | **Interpretation** |
| --- | --- |
| 0–25 | Low burnout |
| 26–50 | Moderate burnout |
| 51–75 | High burnout |
| 76–100 | Very high burnout |
